# TaWRKY70 transcription factor in wheat *QTL-2DL* regulates downstream metabolite biosynthetic genes to resist *Fusarium graminearum* infection spread within spike

**DOI:** 10.1038/srep42596

**Published:** 2017-02-15

**Authors:** Udaykumar Kage, Kalenahalli N. Yogendra, Ajjamada C. Kushalappa

**Affiliations:** 1 Plant Science Department, McGill University, 2111 Lakeshore road, Sainte Anne De Bellevue, Quebec, Canada H9X3V9

## Abstract

A semi-comprehensive metabolomics was used to identify the candidate metabolites and genes to decipher mechanisms of resistance in wheat near-isogenic lines (NILs) containing *QTL-2DL* against *Fusarium graminearum (Fg)*. Metabolites, with high fold-change in abundance, belonging to *hydroxycinnamic acid amides (HCAAs*): such as coumaroylagmatine, coumaroylputrescine and *Fatty acids*: phosphatidic acids (PAs) were identified as resistance related induced (RRI) metabolites in rachis of resistant NIL (NIL-R), inoculated with *Fg.* A WRKY like transcription factor (TF) was identified within the *QTL-2DL* region, along with three resistance genes that biosynthesized RRI metabolites. Sequencing and *in-silico* analysis of *WRKY* confirmed it to be wheat *TaWRKY70*. Quantitative real time-PCR studies showed a higher expression of *TaWRKY70* in NIL-R as compared to NIL-S after *Fg* inoculation. Further, the functional validation of *TaWRKY70* based on virus induced gene silencing (VIGS) in NIL-R, not only confirmed an increased fungal biomass but also decreased expressions of downstream resistance genes: *TaACT, TaDGK* and *TaGLI1,* along with decreased abundances of RRI metabolites biosynthesized by them. Among more than 200 FHB resistance QTL identified in wheat, this is the first QTL from which a TF was identified, and its downstream target genes as well as the FHB resistance functions were deciphered.

Fusarium head blight (FHB) is one of the major constraints in wheat and barley production. Several methods have been used to manage FHB in wheat, among which the use of FHB resistant cultivars is considered to be the most efficient, economic and environmental friendly method^1^. More than 200 QTL have been identified, including a total of 52 QTL associated with rachis resistance based on single floret inoculation^2^. Among these, the *QTL-2DL* is one of the major and the most stable QTL across different genetic backgrounds and various environments^2^. This was first identified from Wuhan-1, a Chinese genotype, in which it explained up to 28% of the total phenotypic variation^3^. However, the genetic determinants undelying these QTL still remain largely unknown. Thus, the identification and functional elucidation of genes from these QTL are very important for their use in breeding.

A recent transcriptomic study of NILs containing *QTL-2DL* attempted to identify candidate genes, but failed to identify any QTL specific genes for resistance to FHB^4^. Apart from this, plentiful transcriptomics and metabolomics studies reported numerous differentially expressed genes and accumulation of metabolites involved in FHB resistance but none of them were validated for gene functions^5^,^6^,^7^,^8^,^9^,^10^,^11^ except for *TaACT* gene in *QTL-2DL*^12^. Therefore, functional analysis of mapped QTL using alternative disciplines like metabolomics integrated with genomics is considered as one of the best tools to decipher the functions of underlying genes. Semi-comprehensive metabolite profiling of barley^13^,^14^ and wheat^7^,^8^,^13^,^14^,^15^,^16^ genotypes with varying levels of resistance to FHB has led to the identification of several RR metabolites and their role in resistance. Recently a semi-comprehensive metabolomics study of barley genotypes, resistant and susceptible to FHB identified a transcription factor *HvWIN1* that regulated downstream resistance genes to biosynthesize fatty acids that were deposited to reinforce cuticle to contain *Fg* infection^17^. In potato, not only the RR metabolites against *Pytophthora infestans* but also their biosynthetic genes were identified^18^,^19^ and functionally validated^20^. Integrated transcriptomics and metabolomics have revealed induction of hierarchies of resistance genes and differential accumulation of defense related metabolites in potato against late blight^21^. The resistance in plants against biotic stress is considered to be due to hierarchies of resistance (*R*) genes with regulatory roles such as elicitor/effector recognition receptors (*R*_*ELRR*_ and/or *R*_*ERR*_), phytohormone biosynthetic genes (*R*_*PHR*_), mitogen-activated protein kinase (*R*_*MAPK*_), and transcription factors (*R*_*TF*_) which regulate the metabolic pathway network genes that biosynthesize resistance related metabolites (*R*_*RRM*_) and/or RR proteins (*R*_*RRP*_) to suppress or contain the pathogen to initial infection^22^.

The WRKYs are one of the largest families of transcriptional regulators in plants and are involved in biotic and abiotic stress responses such as metabolite biosynthesis, cell wall formation, senescence, trichome development, and hormone responses^23^,^24^,^25^,^26^,^27^. WRKY proteins have either one or two WRKY DNA binding domains with a consensus amino acid sequence, WRKYGQK at N-terminal end and a zinc-finger motif at their C-terminal end^25^,^28^. The WRKY TFs regulate target genes by binding to the specific DNA sequence motif (T)TGAC(C/T), which is known as the W-box^28^. WRKYs may be positive or negative regulators of downstream defense mechanisms^25^. For example, WRKY TFs regulates the production of a variety of phenolic-based compounds including lignin^23^,^29^,^30^. Knocking down of *StWRKY1* in potato compromised resistance to *P. infestans* due to reduced accumulation of hydroxycinnamic acid amides^20^. OsWRKY45 is a positive regulator of terpenes such as momilactone, phytocassane, and oryzalexin accumulation, which are involved in plant defense against pathogens and herbivores by activating biosynthetic gene expression^31^. Silencing of *TaWRKY53* in wheat has confirmed its role in aphid defense^32^. Two *WRKY* genes, *NaWRKY3* and *NaWRKY6* coordinate defense response against herbivory in tobacco^33^. *AtWRKY33* is known to regulate biosynthesis of camalexin, and a positive regulator of resistance against the necrotrophic fungi *Botrytis cinerea* and *Alternaria brassicicola*^34^. Contrastingly, *AtWRKY38* and *AtWRKY62* are negative regulators of basal resistance to *Pseudomonas syringae*^35^. Over expression of *AtWRKY48,* showed negative effects towards resistance to *P. syringae*^36^. Similarly, HvWRKY1 and HvWRKY2 are negative defense regulators of powdery mildew resistance in barley^37^. *Gossypium hirsutum (Gh*) WRKY25 negatively regulates *B. cinerea* infection in transgenic tobacco^38^. Over expression of *GhWRKY27a* reduced resistance to *Rhizoctonia solani* infection in tobacco^39^. *AtWRKY50/51* involved in enhanced resistance to *A. brassicicola* but at the same time it also involved in increased susceptibility to *B. cinerea* by mediating salicylic acid and low oleic acid dependent repression of jasmonic acid signaling^40^. In rice, overexpression of *OsWRKY13* up-regulated the phenylpropanoid pathway genes and at the same time down-regulated those involved in terpenoid biosynthesis, illustrating the important role of WRKYs in differential regulation of diverse metabolite biosynthetic genes involved in plant defense^41^. This shows that the WRKYs are of great interest to reveal diverse biotic stress responses in plants.

In wheat, despite huge efforts the elucidation of molecular functions of WRKY genes is still limited. There are at least 200 WRKY genes in wheat but unfortunately only a few of them were studied in detail^42^. Therefore, identification and functional analysis of WRKY TFs in wheat is very important. In this study, we identified and characterized *TaWRKY70* TF from bread wheat. The *TaWRKY70* gene was confirmed to be located within the *QTL-2DL* region, and imparted resistance against FHB by accumulating PAs and HCAAs through regulation of downstream biosynthetic resistance genes *TaDGK, TaGLI1,* and *TaACT.*

## Materials and Methods

### Plant production and experimental design

The near-isogenic lines (NILs) used here were derived from a cross BW301 X HC374^3^,^43^. The BW301 is FHB susceptible hard red spring wheat line from western Canada, and HC374 is resistant to FHB (derived from the cross Wuhan1 x Nyubai). The NILs were genotyped with microsatellite markers. Homozygous lines with susceptible background differing only in the alleles of the *QTL-2DL* locus and did not have any other known FHB resistance QTL located on chromosomes 3B, 4B, 5A and 6B were used to derive the NILs^44^. The seeds of NILs with FHB susceptible and resistant alleles of *QTL-2DL* were obtained from Dr. McCartney, AAFC, Winnipeg, Canada. The experiment was laid out in a randomized complete block design (RCBD) with two genotypes (resistant and susceptible NILs), two inoculations (pathogen and mock-solution) and five biological replications over time with nine plants in three pots as experimental units. The plants grown in greenhouse were maintained at temperature 23 ± 2 °C, photoperiod of 16 h, and relative humidity 70 ± 10%, throughout the growing period. A complex slow releasing fertilizer 14:14:14 (NPK) at the rate of 5 g per pot and 0.03% of trace elements was applied every 15 days to each pot.

### Pathogen production and inoculation

The *Fg* isolate (GZ-3639, obtained from Dr. R. H. Proctor, USA) was grown on potato dextrose agar at 26 °C for four days. For spore production, *Fg* was further sub-cultured on Rye B agar media and kept inverted by exposing the plates to near UV light for three days. From a seven day old culture macroconidia were harvested and spore count was adjusted to 1 × 10^5^ macroconidia ml^−1^ using a hemocytometer (American Scientific Products, USA)^13^. The experimental units consisted of at least 10 spikes per replication selected from three pots containing three plants in each. Three alternate pairs of wheat spikelets at 50% anthesis stage were point inoculated with 10 μl of either macroconidial suspension or mock-solution using a syringe (GASTIGHT 1750 DAD, Reno, USA). Plants were covered with transparent plastic bags sprayed with water to maintain high humidity and the bags were removed at 48 hours post inoculation (hpi).

### Sample collection, metabolite analysis using liquid chromatography-high resolution mass spectrometry (LC-HRMS) and data processing

At 72 hpi, ten spikes for each replicate were harvested and the spike region with three inoculated and three alternate un-inoculated pairs of spikelets was retained. Spikelets (10 × 6 = 60 pairs) from rachis (10 pieces) were separated, and both the samples were frozen immediately in liquid nitrogen and separately stored at −80 °C until further use. Metabolites were extracted from rachis samples in 60% ice cold aqueous methanol. The 5 μl of clear sample extract was used for metabolite analysis based on LC-HRMS (at IRCM, Montreal, Canada) as previously described^13^.

The LC-HRMS output Xcalibur RAW files were converted into mzXML format. The data was analyzed using MZMine2, and the peaks were identified as metabolites based on monoisotopic mass and fragmentation match with databases and available literature^7^,^13^,^19^,^20^. The relative peak intensities of monoisotopic masses of metabolites were subjected to Students *t-*test (SAS v 9.3) in pair wise treatment combinations (RP vs RM, RM vs SM, SP vs SM and RP vs SP, where RP = resistant NIL inoculated with pathogen, RM = resistant NIL inoculated with mock-solution, SP = susceptible NIL inoculated with pathogen, SM = susceptible NIL inoculated with mock-solution) to identify treatment significant metabolites. The abundances of peaks significant at *P* < 0.05^45^, and false discovery rate threshold of 0.05^46^ were retained. False discovery rate of peaks depends mainly on the signal/noise (S/N) ratio; lower the ratio higher is the false discovery rate. Therefore, S/N ratio was kept high to avoid any false discovery^17^. The metabolites, significantly higher in abundance in resistant than susceptible NIL were considered as resistance related (RR) metabolites. Further, these metabolites were grouped into RR constitutive (RRC = RM > SM) and RR induced (RRI = (RP > RM) > (SP > SM)) metabolites. The fold change (FC) in abundance of metabolites in NIL-R was calculated relative to NIL-S (NIL-R/NIL-S)^7^. Only the highly significant and high FC RRI metabolites were prioritized to increase the probability to identify the most effective resistance candidate genes.

### Disease severity and fungal biomass assessment

To evaluate rachis resistance in wheat genotypes, two NILs with resistant and susceptible alleles were planted in RCBD with three biological replications each with three pots sown at three day intervals. Ten spikes were selected, and in each one pair of spikelets in the mid region was inoculated with *Fg* to assess the spread of pathogen from the inoculated spikelet to other through rachis. Plants were covered with transparent plastic bags sprayed with water to maintain high moisture and the bags were removed at 48 hpi. Observations on the number of spikelets diseased were taken at three day intervals until 15 days post inoculation (dpi). Dark brown discolored and/or bleached spikelets were considered as diseased. Disease severity in NILs was quantified as proportion of spikelets diseased (PSD) in a spike, from which the area under the disease progress curve (AUDPC) was calculated^8^. Data was analyzed for significance based on ANOVA using SAS program (SAS v 9.3).

A separate experiment was conducted to assess resistance based on fungal biomass. The experiment was conducted as RCBD with two NILs with two inoculations (pathogen or mock) and three biological replications with two pots each containing three plants. At 50% anthesis stage, five spikes were selected and three alternate pairs of spikelets were point inoculated with 10 μl of either macroconidial suspension in water or mock-solution using a syringe (GASTIGHT 1750 DAD, Reno, USA). After inoculation, plants were covered with polyethylene bags sprayed with water and bags were removed at 48 hpi. The rachis regions containing six pairs of spikelets were harvested at six dpi and immediately frozen in liquid nitrogen and stored at −80 °C until further use. The genomic DNA was extracted and the fungal biomass was quantified using a real-time qPCR by measuring relative copy number of fungal housekeeping gene *Tri6*. The abundance of this gene was normalized with *TaActin*. The relative gene copy number of *Tri6* based on real-time qPCR was used to estimate the amount of fungal biomass^47^. Statistical significance was calculated using Students *t-*test.

### Candidate gene identification based on high fold-change RR metabolites and their physical localization within *QTL-2DL*

The RRI metabolites with high FC in abundance were mapped on to metabolic pathways to find their catalytic enzymes and the coding genes, which were identified using genomic databases (such as KEGG, MetaCyc, PlantCyc and Arabidopsis Acyl metabolic pathways) and available literature. Presence of SSR markers, wmc245, gpw8003, gwm539 and gwm608, were used to define the interval for *QTL-2DL*. Some flanking marker sequences available at GrainGenes database were retrieved, and if not available, they were sequenced in our lab (gpw8003, gwm539 and gwm608). Flanking marker sequences were subjected to BLAST^48^ search in IWGSC chromosome survey sequence repository (Wheat CSS genome reference v2) to physically localize the markers and to define the *QTL-2DL* interval temporarily. Later, the candidate genes identified based on high FC RRI metabolites were BLAST searched to check their co-localization within the temporarily mapped *QTL-2DL* region. Further, this was confirmed by gene prediction using the 2DL chromosome arm sequence from the IWGSC (Chromosome arm sequence assemblies) between the two flanking makers (wmc245 and gwm608). Contigs identified as the best hit for candidate genes were retrieved from database and the gene prediction was performed using SoftBery–FGENESH (http://linux1.softberry.com/berry.phtml?topic=fgenesh&group=programs&subgroup=gfind) program to study the gene structure. The identified gene was amplified using gene specific primers designed using NCBI Primer-BLAST tool (http://www.ncbi.nlm.nih.gov/tools/primer-blast/). Gene prediction and synteny mapping was also performed with rice and brachypodium to predict and locate other putative genes in the *QTL-2DL* region.

### Gene cloning, sequencing and sequence analysis

The genomic DNA was isolated and the full length *TaWRKY70* gene was amplified using primer pairs TaWRKY_F and TaWRKY_R from NILs. Gene amplification was conducted using a thermal cycler (Bio-Rad, Mississauga, ON, Canada) with the following steps: Initial denaturation at 95 °C for 5 min followed by 35 cycles of 94 °C for 30 s, 55 °C for 1 min, 72 °C for 2 min followed by a final extension at 72 °C for 10 min. PCR products were separated on a 1% agarose gel. A band size corresponding to ~1300 bp was then purified from the gel, cloned into the pGEM®-T Easy vector (Promega, USA), and sequenced using the ABI Automated DNA sequencer. DNA sequences were translated to amino acid sequences using the ExPASy Translate Tool (http://web.expasy.org/translate/). The MOTIF Search tool (http://www.genome.jp/tools/motif/) was used to search for functional domains present in deduced amino acid sequence. Further, these results were confirmed using PROSITE tool (http://www.expasy.ch/prosite) and NCBI Conserved Domain Database (NCBI CDD). The multiple sequence alignment was performed using MultAlin (http://multalin.toulouse.inra.fr/multalin/) and maximum-likelihood phylogenetic relationships were determined using Phylogey.fr (http://www.phylogeny.fr/) program.

### RNA isolation and gene expression based on qRT-PCR

For relative quantification of transcript expression, the total RNA of rachis was isolated from five biological replicates using RNeasy plant mini kit (Qiagen Inc.). Purified total RNA (1–2 μg) was used to reverse transcribe RNA into cDNA using iScript cDNA synthesis kit (BioRad, ON, Canada). Using equal quantity of cDNA (20 ng) for each sample, real-time qRT-PCR was performed using Qi SYBR Green supermix (BioRad, Canada) in a CFX384TM Real-Time system (BioRad, Canada). The mRNA abundance of target gene was normalized with *TaActin* transcript level. PCR results were analyzed using comparative delta-delta Ct method (2^−ΔΔCT^)^49^. The statistical significance of observations was analyzed based on Students *t-*test.

### Nuclear localization assay

The LocSigDB (http://genome.unmc.edu/LocSigDB/) was used for nuclear localization signal (NLS) prediction. The full-length coding region of *TaWRKY70* was amplified and cloned into pCX-DG vector containing green florescence protein (GFP) and *Cauliflower Mosaic Virus (CaMV*) 35S promoter^50^. For subcellular localization study, TaWRKY70 + GFP fusion and GFP alone (as a control) were transfected into potato protoplasts using a polyethylene glycol-calcium method^51^. Transfected protoplasts were incubated at 23 °C for 16 h and analyzed for GFP florescence by florescence microscopy. This experiment was conducted three times.

### Luciferase (LUC) transient expression assay

The coding region of the *TaWRKY70* gene and the promoters of *TaACT, TaDGK* and *TaGLI1* from the resistant genotype were amplified, cloned into pGEM®-T Easy vector (Promega, USA), and confirmed by sequencing. This was followed by sub-cloning into the in FU63 (CD3-1841) vector^52^. For LUC transient expression assays, reporter plasmids (ACTp-LUC or DGKp-LUC or GLI1p-LUC or vector control having 30 bp DNA fragment without w-box), effector constructs containing *TaWRKY70*, and 35 S::β-glucuronidase (GUS) internal control were co-transformed into potato protoplasts. The protoplasts were pelleted and re-suspended in 1× cell culture lysis reagent (Promega, USA). GUS fluorescence was measured using a Modulus luminometer/fluorometer with a UV fluorescence optical kit (Fluorescence Microplate Reader; BioTek, USA). The experiment was carried out in three replicates; each replicate contained 20 μl protoplast lysate and 100 μl LUC mix. LUC activity was detected with a luminescence kit using LUC assay substrate (Fluorescence Microplate Reader). The relative reporter gene expression levels were expressed as LUC/GUS ratios, which were used to discriminate treatments. The significance between treatments and vector control was analyzed using students *t-*test at *P* < *0.01*.

### Construction of BSMV vectors and virus induced gene silencing of *TaWRKY70*

For transient gene silencing, 283 bp fragment of *TaWRKY70* gene was selected with efficient siRNA generation and no off-target genes into the modified viral genome using siRNA Scan tool (http://bioinfo2.noble.org/RNAiScan.htm), and a BLAST search of fragment against GenBank database. We chose the most divergent sequence containing (3′ UTR region) to increase the specificity (Fig. S1a). The fragment was amplified from cDNA using the primers listed in Table S1. Fragment was cloned into the pGEM®-T Easy Vector (Promega Corp., WI, USA) and sequence was confirmed. Plasmid DNA was digested using *NotI* (New England Biolabs, MA, USA), thereby generating *NotI* ends in DNA fragment. The cDNA fragment was subsequently ligated to pSL038-1 vector a plasmid encoding a modified BSMV γ genome segment with a *NotI* cloning site downstream of the γb gene^53^ (Fig. S1b and c). The pSL038-1 vector carrying either *phytoene desaturase* (PDS) or without any gene served as positive and negative controls respectively. The plasmids BSMV α, pγSL038-1 were linearized with *Mlu1* restriction enzyme whereas; BSMV β was linearized by using *Spe1* enzyme. Linearized plasmids were converted into capped *in-vitro* transcripts using mMessage Machine™ T7 *in-vitro* transcription kit (Ambion, Inc., Austin, TX, USA), following the manufacturer’s protocol.

The experiment was designed as a RCBD with one genotype the resistant NIL, two treatments of silenced or non-silenced with five biological replicates over time each with two pots. Plants produced as detailed earlier were rub-inoculated with all the three *in-vitro* transcript reactions (α, β and γ BSMV) in 1:1:1 ratio (1 μl of each) along with 22.5 μl inoculation buffer (1% sodium pyrophosphate, 1% bentonite, 1% celite in 0.1 M glycine, 0.06 M dipotassium phosphate)^54^ that facilitated viral entry and infection. To increase the silencing efficiency both the flag leaf and spikelets were rub-inoculated^55^. The experimental units consisted of five plants from two pots with a total of ten spikes per replicate which were separately rub-inoculated with test (*BSMV* + *TaWRKY70*) and negative control (*BSMV:00*). The flag leaves and spikelets rub inoculated with *BSMV* + *PDS* were served as positive controls.

### Confirmation of gene silencing by qRT-PCR, estimation of fungal biomass and abundances of targeted metabolites

At 12 dpi with virus, three alternate spikelets were inoculated with 10 μl of *Fg* spore suspension and covered with water sprayed plastic bags. The bags were removed at 48 hpi and five out of ten spikes were collected at 72 hpi for qRT-PCR and metabolite analysis in rachis. Similarly, at six dpi, remaining spike samples were collected for the relative quantification of fungal biomass in rachis as relative gene copy number of the fungal housekeeping gene *Tri6* over *TaActin* housekeeping gene.

## Results

### Disease severity and fungal biomass in spikelets and rachis of NIL

The disease severity in spikelets of NILs, with alternate alleles for resistance at *QTL-2DL* with one pair of mid spikelets inoculated with *Fg* was assessed as proportion of spikelets diseased in a spike (PSD), from which the AUDPC was calculated. The AUDPC was significantly higher in NIL-S (2.33) compared to NIL-R (1.48), with a FC = 1.57. The rachis resistance in NILs was assessed based on the amount of fungal biomass in the rachis regions containing six pairs of spilelets, where three alternate pairs were inoculated with *Fg*. The fungal biomass estimated at 6 dpi as relative gene copy number of *Tri6* normalized to *TaActin* based on real-time qPCR was also significantly higher (5.8 FC) in NIL-S than in NIL-R (Kage *et al*.^12^). This clearly demonstrated a high level of rachis resistance associated with *QTL-2DL* against FHB.

### Metabolite profiles of NILs

Semi-comprehensive metabolomics of rachis samples collected at 72 hpi identified several differentially accumulated RR metabolites in NILs with contrasting alleles at *QTL-2DL* for resistance against *Fg*. The significant metabolites were categorized into RRC and RRI. The RRI metabolites with high FC in abundance mainly belonged to two chemical groups: (*i) phosphatidic acids and derivatives* (PAs): [(1-heptadecanoyl,2-(5Z,8Z,11Z,14Z-eicosatetraenoyl)-sn-glycero-3-phosphat = PA(17:0/20:4(5Z,8Z,11Z,14Z) (FC = 54.3)), (1-pentadecanoyl-2-(8Z,11Z,14Z-eicosatrienoyl)-glycero-3-phosphate = (PA(15:0/20:3(8Z,11Z,14Z)(FC = 9.5)) and (1-(9Z-nonadecenoyl)-2-(13Z,16Z-docosadienoyl)-glycero-3-phosphate = (PA(19:1(9Z)/22:2 (13Z,16Z)(FC = 2.3))]; (*ii) hydroxycinnamic acid amides (HCAAs*): p-coumaroylagmatine (FC = 28.7), p-coumaroyputrescine (FC = 9.5) (Table 1).

### Identification of candidate genes in *QTL-2DL*

**Figure 1:**
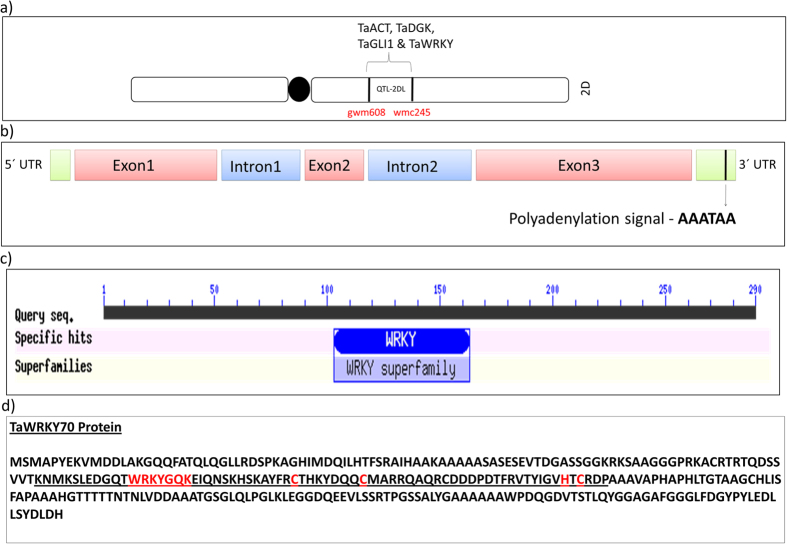
(**a**) Physical map of the *QTL-2DL* on long arm of wheat chromosome 2D. Location of the of candidate genes identified within the *QTL-2DL* region are shown on the right side and flanking markers on the left side of the 2D chromosome; (**b**) Schematic diagram depicting the *TaWRKY70* gene structure containing exon, intron and coding regions; (**c**) Conserved domain predicted based on NCBI Conserved Domain Database Search, it shows presence of conserved WRKY domain; (**d**) Figure showing the characteristic features of Group III WRKY transcription factors, showing the DNA binding domain containing 69 amino acids underlined. It has WRKYGQK and C2HC, WRKY and zinc finger conserved motifs, respectively.

Putatively identified high FC RRI metabolites were mapped on to metabolic pathways and the candidate genes (*R*_*RRM*_) corresponding to the enzymes that biosynthesized these RRI metabolites were identified using the public databases and available literature. The agmatinecoumaroyl transferase (*ACT*) is a rate limiting enzyme in the biosynthesis of HCAAs such as coumaroylagmatine and coumaroylputrescine^56^,^57^. Whereas, the diacylglycerol kinase (*DGK*) and glycerol kinase (*GLI1*) are important enzymes in the biosynthesis of PAs in plants^58^,^59^,^60^,^61^. BLAST analysis positioned the closest gene matches for these enzymes, *ACT, DGK* and *GLI1* within the presumed interval of *QTL-2DL* (Fig. 1a). The genes (*TaACT* and *TaDGK*), including their promoters were sequenced and the sequences were deposited in NCBI database. Sequence comparison of *TaACT* and *TaDGK* in contrasting NILs revealed that only the *TaACT* was polymorphic but not the *TaDGK*. This led us to suspect a possible involvement of TF in regulating PAs pathway. While searching for other candidate genes, coincidentally a WRKY like TF was found in the *QTL-2DL* region based on gene prediction. A list of other predicted genes present in *QTL-2DL* identified based on synteny study with rice and brachypodium are given in Supplementary Data (Table S2), but their roles in FHB resistance are yet to be confirmed.

### *TaWRKY* gene sequencing and sequence analysis

**Figure 2:**
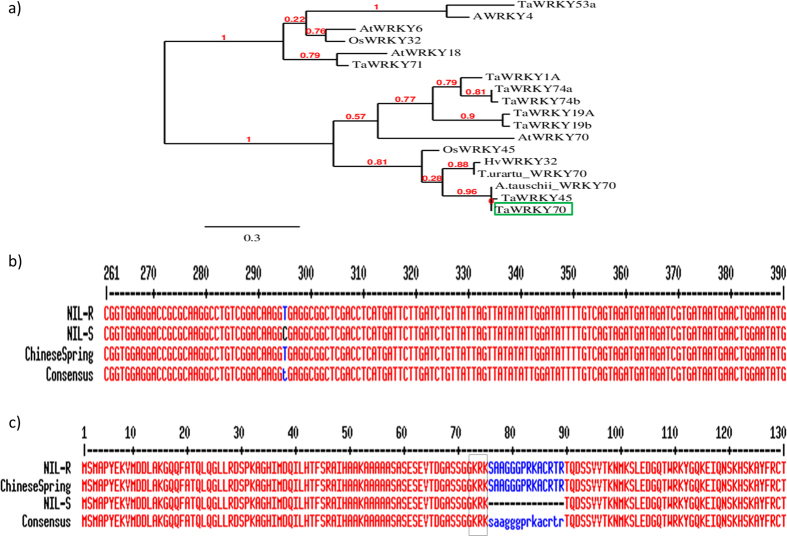
(**a**) Phylogenetic relationships of *TaWRKY70* (in green box) with other plant *WRKY* sequences obtained from NCBI database for wheat (*TaWRKY1A, TaWRKY19A, TaWRKY74a, TaWRKY53a, TaWRKY71, TaWRKY74b*, and *TaWRKY19b*), rice (*OsWRKY45* and *OsWRKY32*), arabidospsis (*AtWRKY18, AtWRKY6, AtWRKY70* and *AtWRKY4*), barley (*HvWRKY32*), *Aegilops tauschii (WRKY70*), *Triticum urartu (WRKY70*). Neighbor-joining tree representing relationships among WRKY proteins from different plant species. Numbers in red color represents branch length; (**b**) Comparison of DNA sequence variation between NIL-R, NIL-S and Chinese spring *TaWRKY70.* It shows a single nucleotide polymorphism at 294 bp position; (**c**) Comparison of protein sequence variation between NIL-R, NIL-S and Chinese spring TaWRKY70. Box shows the predicted nuclear localization signal. It shows 14 amino acids deletion in NIL-S as a result of SNP at 294 bp in the DNA sequence causing shift in splice site.

The WRKY TFs are known to be involved in regulating plant responses to biotic and abiotic stresses. Therefore, we sequenced the predicted full length *WRKY* gene from the genomic DNA of NILs. Sequence analysis based on FGENESH suggested that *WRKY* has three exons and two introns (Fig. 1b) and the intron-exon boundaries were confirmed to be AG and GT at the acceptor and donor sites respectively through FSPLICE (http://linux1.softberry.com/berry.phtml?topic=fsplice&group=programs&subgroup=gfind). The full length sequence of *TaWRKY* was 1288 bp in length containing an open reading frame (ORF) of 1165 bp, a 96 bp 3′ untranslated region (UTR) and 27 bp 5′ UTR (Fig. 1b). One of the plant canonical polyadenylation signals, a six-nucleotide near-upstream element (NUE - AAATAA) was found in the 3′ UTR at the position 1251 to 1257 bp (Fig. 1b)^62^. The complete genomic sequence was submitted to NCBI and was assigned a GenBank accession number KU562861. The putative protein encoded by *TaWRKY* consisted of 290 amino acids. It has conserved characteristic DNA-binding domain comprising a single WRKY domain and Cys2-His-Cys type zinc-binding motif spanning from position 98 to 166 amino acids (Fig. 1c and d). The Group III WRKYs differs from groups I and II in its altered C2-HC zinc finger motif C-X7-C-X23-HX-C^63^ (Fig. 1d). Multiple sequence alignment and phylogenetic analyses indicated that the *TaWRKY* belonged to Group III type of WRKY family (Fig. 2a). The *TaWRKY* showed 99% identity with *Aegilops tauschi* putative *WRKY70* orthologues of *AtWRKY70* and wheat *WRKY45*. Recent studies have placed several WRKY TFs, including AtWRKY70, downstream of NPR1 in the SA signaling pathway on the basis of transcriptional profiling using Arabidopsis npr1 mutant plants^64^. Whereas, rice WRKY45 which is orthologue to AtWRKY70 acts under SA pathway but independent of NH1 (orthologue of AtNPR1)^65^. Therefore, it was designated as OsWRKY45 but not OsWRKY70^65^. Based on this we putatively designated *TaWRKY* as *TaWRKY70* like TF gene (or as *TaWRKY70*) since we are not sure about the position of wheat WRKY45/70 in SA pathway.

### Sequence variation of *TaWRKY70* between NILs and differential gene expression during *Fg* infection

Multiple sequence alignment of *TaWRKY70* gene between NILs and *T. aestivum* cv. Chinese Spring revealed single nucleotide polymorphisms (SNP) in NIL-S at 294 bp position (Fig. 2b), which is exactly at the first exon-intron junction. This resulted in 14 amino acid deletion in predicted protein sequence by shifting open reading frame (ORF) upstream to its normal, causing a lack of 42 bp sequence in the first exon of the *TaWRKY70* transcript (Fig. 2c). Further the deleterious effect of these SNPs on protein functionality was confirmed based on *in-silico* analysis by Phyre 2 Investigator (data not shown). However, additional experimental proofs are needed to confirm the mutation and to know how it induces the truncated protein *in-vivo* and alters the transcriptional activity of TaWRKY70 ultimately affecting resistance against *Fg*.

**Figure 3:**
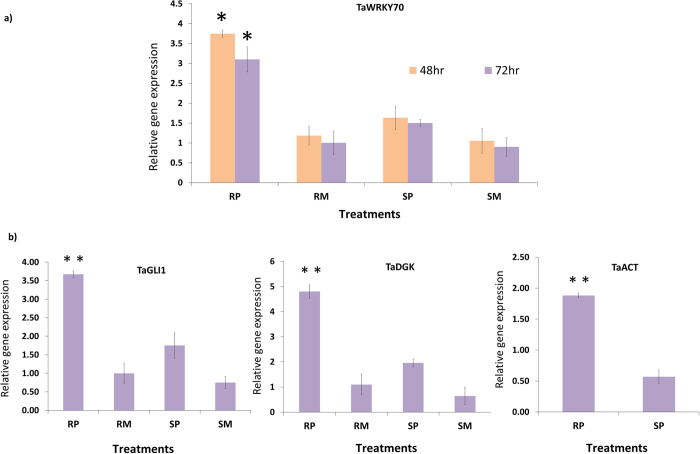
Relative transcriptional changes of *TaWRKY70* and its downstream genes induced by *Fg* and mock (water) inoculation based on qRT-PCR. Here target gene expression is normalized to reference gene *TaActin.* (**a**) Relative transcriptional changes of *TaWRKY70* at 48 and 72 hpi; (**b**) Gene expression of *TaDGK* and *TaGLI1* and *TaACT* (which did not show any expression in mock treated samples) at 72 hpi. RP = Resistant pathogen, RM = Resistant mock, SP = Susceptible pathogen and SM = Susceptible mock. Significant differences in expression levels of RP as compared to SP using Students *t*-test: *P < 0.05; **P < 0.01.

The relative gene expression of *TaWRKY70* following *Fg* inoculation was significantly (*P* < 0.05) higher (2.3 FC at 48 hpi and 2.0 FC at 72 hpi) in pathogen treated NIL-R compared to pathogen treated NIL-S. Similarly, its expression was higher in pathogen treated NIL-R compared to mock treated samples of both the NILs at both the time points (48 hpi and 72 hpi) though the expression levels were slightly lower at 72 hpi (Fig. 3a), suggesting *TaWRKY70* has a potential role against *Fg* resistance in the early stages of defense through activation of downstream genes.

### Gene expression, promoter analysis of RR metabolite biosynthetic genes and their physical interaction with *R*_*TaWRKY70*_

**Figure 4:**
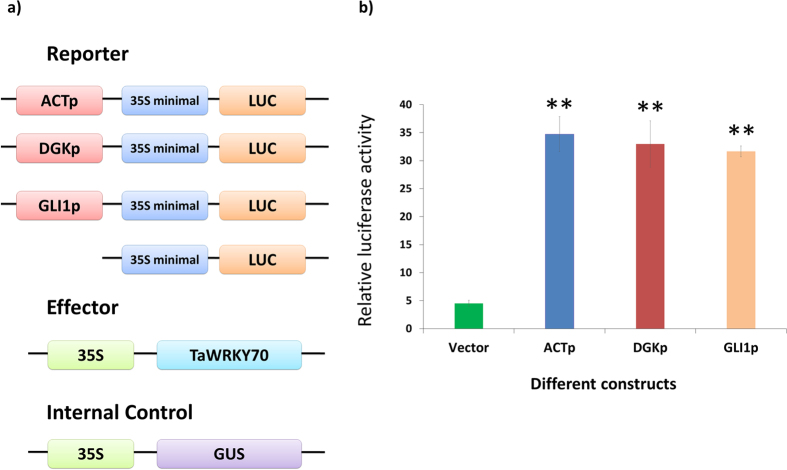
Transcriptional regulation of RRI metabolite biosynthetic genes by *TaWRKY70*. (**a**) Constructs used in the transient expression assay and (**b**) relative luciferase (LUC) reporter activity by *TaWRKY70*. The relative reporter gene expression levels were expressed as LUC/GUS ratios. Values are averages of three replicates. Significant differences in expression levels in promoters compared with vector based on Student’s *t*-test: **P < 0.01.

At 72 hpi, the relative gene expression levels of downstream *R*_*RRM*_ genes: *TaDGK* (2.4 FC), *TaGLI* (2.0 FC), and *TaACT* (3.3 FC) were significantly (*P* < 0.01) higher in NIL-R than in NIL-S after *Fg* inoculation; the trend was similar with *TaWRKY70* (Fig. 3b). To further study the downstream *R*_*RRM*_targets of *TaWRKY70*, we performed promoter analysis of *R*_*TaACT*_*, R*_*TaDGK*_ and *R*_*TaGLI1*_. The promoter sequences from −1 bp to −1000 bp upstream of ATG start site were considered for analysis using a PLACE database (http://www.dna.affrc.go.jp/PLACE/) and manual search. The promoter analysis of these genes revealed the presence of putative W-box sequence in their promoters within −500 bp (Table 2). Further we confirmed the potential interaction of *TaWRKY70* with these downstream *R*_*RRM*_ genes using *Arabidopsis* as a search organism in GeneMANIA software (http://www.genemania.org/). Resulting networks showed a clear interaction between *TaWRKY70* and the *R*_*RRM*_ genes (*TaACT, TaDGK* and *TaGLI1*) (Fig. S2). The physical interaction *in-vivo* was confirmed based on luciferase assay. The reporter and effector constructs were transformed into potato protoplasts to check the expression of LUC reporter. We found drastic increase in the expression of LUC reporter in *TaACT* (34.7), *TaDGK* (32.9) and *TaGLI1* (31.6) promoters as compared with vector alone (4.5) (Fig. 4a,b), suggesting *TaWRKY70* regulates transcription of *TaDGK, TaGLI1* and *TaACT* to biosynthesize PAs and HCAA, thus confirming their *in-silico* predicted interaction.

### Nuclear localization of TaWRKY70 protein

**Figure 5:**
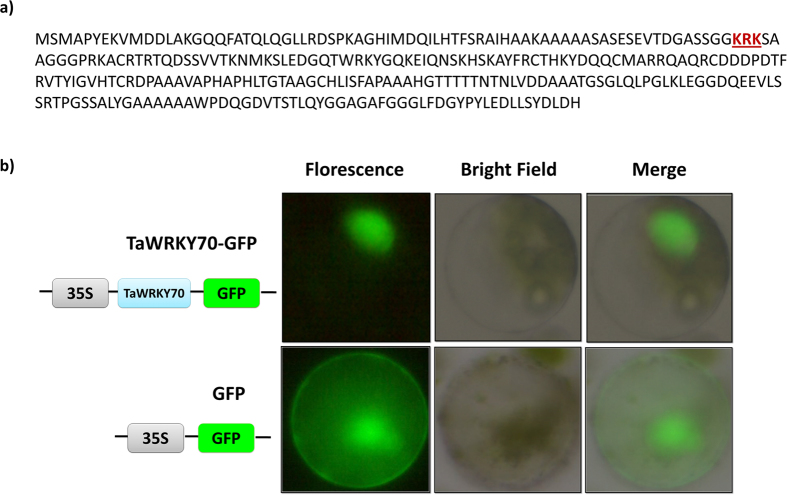
Nuclear localization of TaWRKY70 protein. (**a**) Nuclear localization signal (NLS) predicted. Red colored amino acid region in bold font and underlined is a NLS; (**b**) Nuclear localization analysis. Constructs consisting of either TaWRKY70-GFP fusion or GFP alone were used to transiently transform into potato protoplasts. Free GFP and TaWRKY70-GFP fusion proteins were transiently expressed in potato protoplast and observed with a fluorescence microscope. Here, the extreme left panel (GFP fluorescence), the middle panel (bright field) and the right panel (merged view of two images). Transient expression assays were conducted at least three times.

To investigate the subcellular localization of TaWRKY70 protein, we used LocSigDB (http://genome.unmc.edu/LocSigDB/) with default setting. We found three conserved amino acids region (KRK) potentially acting as NLS for TaWRKY70 protein (Fig. 5a). Additionally, we used transient expression system in potato protoplasts to characterize the subcellular localization of TaWRKY70 protein. This demonstrated that TaWRKY70 + GFP fusion protein was localized in the nucleus, while the control vector (GFP alone) was expressed in the cytosol and nucleus (Fig. 5b). These results agreed with the subcellular localization prediction, suggesting that the TaWRKY70 is a nuclear protein.

### Response to *Fg* infection after knocking down of *TaWRKY70* in wheat

Based on the changes in *TaWRKY70* expression after *Fg* inoculation, the BSMV-VIGS system was employed to knock down the transcription of *TaWRKY70* and to further investigate its function in response to *Fg* infection. The feasibility and silencing efficiency of the BSMV-VIGS system in NIL-R was tested using the wheat phytoene desaturase (*TaPDS*) as a positive control. At 12 dpi with *BSMV:TaPDS*, photo-bleaching symptoms started appearing on wheat spikes when *TaPDS* was silenced (Fig. S3). Therefore, the BSMV-VIGS system was used for assessing the potential roles of *TaWRKY70* in wheat resistance against *Fg* infection. Under the same conditions, the *BSMV:TaWRKY70* (test/silenced) and *BSMV:00* (control/non-silenced) recombinant vectors were rub-inoculated onto the NIL-R.

**Figure 6:**
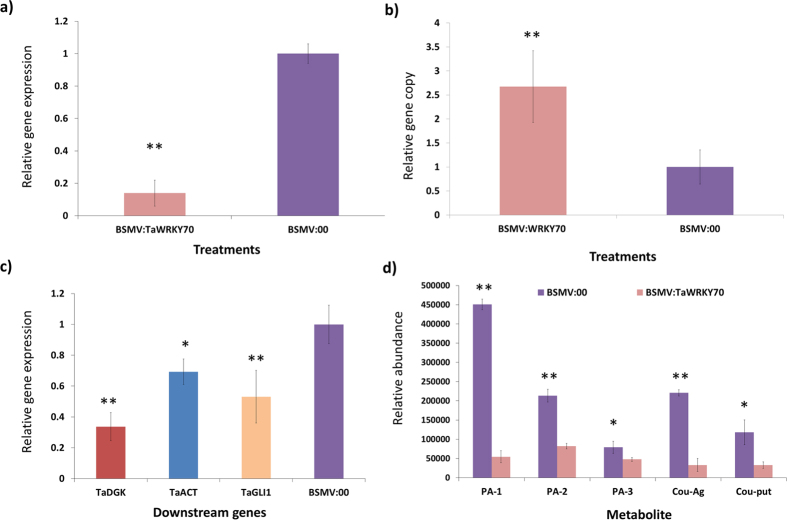
Effect of *TaWRKY70* silencing in FHB resistant near-isogenic line (NIL-R), inoculated with *F. graminearum* or mock-solution. (**a**) Confirmation of knocking down of *TaWRKY70* by assaying relative transcript expression of *TaWRKY70* normalized to reference gene *TaActin* in silenced plant (BSMV:*TaWRKY70*) compared to non-silenced (BSMV:00) at 3 dpi after *Fg* inoculation; (**b**) Fungal biomass in BSMV-infected plants at 6 dpi with *Fg*. Relative copy number of *Tri6* fungal housekeeping gene (=fungal biomass) was quantified in *TaWRKY70* knocked down (BSMV:*TaWRKY70*) plants and compared with control (BSMV:00). Here relative target gene copy number is normalized to reference gene *TaActin*; and (**c**) Relative transcript levels of *TaDGK, TaACT* and *TaGLI1* assayed individually in *TaWRKY70* knocked down (BSMV:*TaWRKY70*) plants compared to non-silenced (BSMV:00) at 3 dpi after *Fg* inoculation. Here target gene expression is normalized to reference gene *TaActin*; (**d**) Relative metabolite abundances of RRI metabolites in silenced (BSMV:*TaWRKY70*) and non-silenced (BSMV:00) NIL-R at 3 dpi after *Fg* inoculation. PA-1 – PA(17:0/20:4(5Z,8Z,11Z,14Z)), PA-2 – PA(15:0/20:3(8Z,11Z,14Z)), PA-3 – PA(19:1(9Z)/22:2(13Z,16Z)), Cou-Ag – p-coumaroylagmatine and Cou-put – p-coumaroylputrescine. Significant differences in expression levels as compared in silenced (BSMV:*TaWRKY70*) with non-silenced (BSMV:00) using Students *t*-test: *P < 0.05; **P < 0.01.

To study the efficiency of silencing of *TaWRKY70* in NIL-R plants that had been infected with recombinant BSMV vectors, the relative expression levels in rachis were detected by qRT-PCR. The relative expression of *TaWRKY70* was significantly (*P* < 0.01) reduced by 86.04% in plants infected with *BSMV:TaWRKY70* compared to *BSMV:00* infected plants at 72 hpi with *Fg* confirming the silencing or down regulation of target gene in wheat rachis (Fig. 6a). To further determine whether silencing of *TaWRKY70* in NIL-R compromised resistance to *Fg* infection, the fungal biomass of *Fg* was estimated by measuring the relative transcript levels of the *Fg* housekeeping gene *Tri6* over *TaActin*. Fungal biomass was significantly higher (*P* < 0.01) in silenced plants as compared to nonsilenced control plants (Fig. 6b). These results suggest that the enhanced susceptible phenotypes observed in NIL-R inoculated with the *Fg* were due to the silencing of *TaWRKY70*.

### Silencing of *TaWRKY70* affected transcriptional response of *R*_*RRM*_ genes and RR metabolite accumulation

It is evident from the confirmation of the presence of W-Box in promoters based on GeneMANIA analysis, and luciferase assay that *TaWRKY70* physically interacts with the downstream targets like *TaACT, TaDGK* and *TaGLI1.* Therefore, to check whether knocking down of *TaWRKY70* has affected the transcriptional responses of downstream candidate genes, the relative expression levels of these genes were estimated using qRT-PCR in silenced samples. Coincidently, the expression levels of *TaACT* (FC = 3.44)*, TaDGK* (FC = 1.36) and *TaGLI1* (FC = 1.88) were significantly down regulated in silenced as compared to non-silenced samples, further confirming these genes as potential interacting targets of *TaWRKY70* (Fig. 6c). Additionally, to confirm the biochemical and molecular mechanisms of the involvement of *TaWRKY70* to resist FHB, metabolite profiling was performed in silenced and non-silenced NIL-R plants. In NIL-R silenced plants, the abundances of candidate RR metabolites such as, *PAs*: (PA(17:0/20:4(5Z,8Z,11Z,14Z)), PA(15:0/20:3(8Z,11Z,14Z)) and PA(19:1(9Z)/22:2(13Z,16Z)) with FC = 8.3, 2.5 & 1.6, respectively), and *HCAAs*: p-coumaroylagmatine (FC = 6.7) and p-coumarolyputrescine (FC = 3.5) were significantly (*P* < 0.01) reduced compared to non-silenced plants (Fig. 6d). This clearly implied the involvement of *TaWRKY70* in the regulation of downstream RR metabolite biosynthetic genes in NIL-R, production of RRI metabolites and an eventual resistance against FHB.

## Discussion

Resistance in plants against pathogen attack is controlled by several hierarchies of resistance genes that eventually biosynthesize resistance related metabolites and proteins that directly suppress and/or contain the pathogen to initial infection through their antimicrobial and/or cell wall reinforcement properties^22^,^66^. Plant *R*_*ELRR*_ genes recognize the pathogen produced elicitors and trigger downstream *R*_*MAPK*_ and *R*_*TF*_genes, which regulate the *R*_*RRM*_and *R*_*RRP*_genes that biosynthesize RR metabolites and proteins. Thus it is crucial to map the network of plant genes involved in the hierarchy to resist the pathogen. Some of these genes have major or minor resistance effects. In this study, we report wheat TF as one of the candidate genes with significant FHB resistance effect, through regulation of several downstream *R*_*RRM*_ genes that biosynthesize RRI metabolites that directly suppress and/or contain pathogen advancement.

Metabolites are the end products of genes, and thus they better represent the phenotype. Accordingly, metabolite profiling was used as a primary tool to explore the *R*_*RRM*_ genes involved in NILs with contrasting levels of FHB resistance at *QTL-2DL*. Only the high FC RRI metabolites were considered to explore the *R*_*RRM*_ genes. The PAs and HCAAs were the major RRI metabolites found in wheat rachis after pathogen invasion. Phosphotidic acid is the essential intermediate for the *de-novo* biosynthesis of all glycerolipids^67^. PAs and their derivatives are basically involved in signaling and structural fortification of the cell wall through deposition of glycerol 3-phosphates^68^,^69^. This also helps in the suppression of cell death induced by hydrogen peroxide^70^. The HCAAs are not only phytoalexins that suppress pathogens due to their antimicrobial activity but also are deposited in the secondary cell walls reinforcing them to contain the pathogen to initial infection^71^. Considering these roles of RRI metabolites identified here, we mapped these metabolites on to their metabolic pathways to identify their biosynthetic genes. Based on available information from databases and literature, the genes involved in biosynthesizing these RRI metabolites identified were: *TaACT, TaDGK* and *TaGLI1*. Several studies have reported the biotic stress resistance roles of *ACT*^56^,^57^,^72^, *DGK*^73^,^74^ and *GLI1*^59^,^75^,^76^. While searching for other genes in the *QTL-2DL* region we found a gene encoding WRKY like protein. The WRKY proteins are regulatory in nature and have a role in plant biotic and abiotic stress resistance by controlling the transcription of downstream *R* genes by binding to W-Box cis-elements present in their promoters^77^. Further, sequence analysis of *TaWRKY70* gene revealed polymorphism between NILs. The SNPs in NIL-S at the position of 294 bp, in the first exon-intron junction, led to a shift in the open reading frame, which resulted in truncated protein. This might affect the protein structure and function, proving which would require additional studies. The levels of gene expression of *TaACT, TaDGK, TaGLI1* and *TaWRKY70* were higher in NIL-R compared to its susceptible counterpart. Further, the disease severity and fungal biomass in NIL-R were significantly lower than in NIL-S, as confirmed in our previous study^12^. Taken together; these results demonstrate the potential roles of these candidate genes in FHB resistance in wheat.

Sequence comparison of *TaDGK* in NILs revealed the absence of sequence variation, both at the coding and promoter regions, and in spite of this the transcript expression levels were higher in NIL-R than in NIL-S. To answer this, we sequenced and analyzed the promoter regions of *TaACT, TaDGK* and *TaGLI1,* which revealed the presence of W-Box *cis-*element in their promoter, giving a clue on the involvement of *TaWRKY70* protein in the regulation of these downstream *R*_*RRM*_genes by binding to their promoter. Subcellular localization study showed *TaWRKY70* to be localized in nucleus. Further, the bioinformatics analysis of protein-DNA interaction networks using GenMANIA software showed that *TaWRKY70* interacts with all the three *R*_*RRM*_genes (*TaACT, TaDGK* and *TaGLI1*) that biosynthesized the candidate RRI metabolites identified here. Further, their physical interaction was proved based on luciferase assay *in-vivo*. These results present compelling evidence on the involvement of *TaWRKY70* in FHB disease resistance, by regulating downstream genes that produced RRI metabolites with signaling, antimicrobial and cell wall reinforcement properties.

Association of RRI metabolites with *R*_*RRM*_genes alone is not enough to claim the role of *TaWRKY70* gene in FHB resistance, and they need to be functionally validated. Among several tools available such as gene mutagenesis, insertional mutagenesis, RNAi and VIGS, the VIGS is considered to be the best tool for its easy and rapid knockdown ability of genes during plant development and also it enables assessment of the lack of resistance effect induction in plant by the pathogen^78^. There are several successful reports on the use of VIGS in functional genomics in tobacco, tomato, Arabidopsis, potato, wheat and barley, as these plants have well established vectors for gene silencing. Therefore, we used VIGS as a tool in functional characterization of *TaWRKY70* in NIL-R. The knocking down of *TaWRKY70* in NIL-R resulted in the reduction of transcript abundance of *TaACT, TaDGK* and *TaGLI1* which in turn decreased the abundances of their biosynthetic RRI metabolites, resulting in increased fungal biomass. The silenced NIL-R phenotype was quite similar to NIL-S phenotype as determined based on the amount of fungal biomass. These results indicate that *TaWRKY70* TF modulates the expression of several *R*_*RRM*_ genes, of which *TaACT, TaDGK* and *TaGLI1* may be a subset. PAs biosynthetic genes were confirmed to be regulated by TFs in *Nannochloropsis* spp., a group of oleaginous microalgae^79^. Knock-down of *FcWRKY70* in kumquat down-regulated *ADC* (arginine decarboxylase) gene expression and decreased putrescine abundance level accompanied by compromised dehydration tolerance^80^. Late blight pathogen infection in potato induced HCAA biosynthetic genes regulated by *StWRKY1*, and the promoter region sequence analysis of 4-coumarate:CoA ligase (*St4CL*) and tyramine hydroxycinnamoyl transferase (*StTHT*) revealed the W-box sequence, demonstrating the WRKY binding activity^20^. Overexpression of *TaWRKY45* has showed enhanced resistance to powdery mildew, leaf rust and fusarium head blight diseases in wheat but mechanisms of resistance were not known^81^. Whitefly infestation in Arabidopsis also induced *AtWRKY* and regulated *At4Cl4* expression by binding to W-box present in its promoter^82^. Similarly, in wheat aphid infestation induced *TaWRKY53,* silencing of which significantly reduced the expression of *PAL* gene^32^ suggesting that the network of these genes are involved in imparting resistance to several biotic stresses.

In summary, we have identified and isolated Group III stress-responsive WRKY gene designated as *TaWRKY70* from wheat, which acts as a positive regulator of resistance against *Fg*. The FHB resistant NIL containing *TaWRKY70* accumulated high amounts of RRI metabolites, whereas the *TaWRKY70* silenced plants had reduced amounts. The *R*_*RRM*_ genes such as *TaACT, TaDGK* and *TaGLI1* along with *R*_*TF*_ gene *TaWRKY70* were localized within the *QTL-2DL* region. Furthermore, the promoter analysis of the candidate *R*_*RRM*_genes, *TaACT, TaDGK* and *TaGLI1* revealed W-box elements and the luciferase assay confirmed their regulation by *TaWRKY70.* Collectively, these results indicated that the *TaWRKY70* gene functioned in mediating FHB resistance by elevating the accumulation of PAs and HCAAs metabolites by regulating downstream *TaACT, TaDGK* and *TaGLI* genes (Fig. S4). A simplified model to explain the interaction of regulatory and RR metabolite biosynthetic genes can be expressed as: *R*_*TaWRKY70*_ * (*R*_*TaACT*_ + *R*_*TaDGK*_ + *R*_*TaGLI1*_) to provide a global view of the mode of action of *TaWRKY70* in FHB resistance. In conclusion, taken together, the *TaWRKY70* gene in the *QTL-2DL* governs major resistance effect against *Fg*. Following further validation, this gene can be used in wheat FHB resistance breeding programs or for genome editing in susceptible commercial cultivars, if these genes are found non-functional to enhance resistance in wheat against FHB (Kushalappa *et al*.^66^).

## Additional Information

**How to cite this article**: Kage, U. *et al*. TaWRKY70 transcription factor in wheat *QTL-2DL* regulates downstream metabolite biosynthetic genes to resist *Fusarium graminearum* infection spread within spike. *Sci. Rep.* **7**, 42596; doi: 10.1038/srep42596 (2017).

**Publisher's note:** Springer Nature remains neutral with regard to jurisdictional claims in published maps and institutional affiliations.

## Supplementary Material

Supplementary Dataset

## Figures and Tables

**Table 1:** List of high fold change resistance related induced (RRI) metabolites identified in NILs with contrasting levels of FHB resistance alleles at *QTL-2DL* inoculated with *F. graminearum* or mock-solution.

RRI	Name	FC	Category
Observed mass			
710.4887	PA(17:0/20:4(5Z,8Z,11Z,14Z))	54.3***	Glycerophospholipids
276.1592	p-Coumaroylagmatine	28.7***	HCAA
234.1373	p-Coumaroylputrescine	9.5**	HCAA
684.4729	PA(15:0/20:3(8Z,11Z,14Z))	9.5**	Glycerophospholipids
768.5699	PA(19:1(9Z)/22:2(13Z,16Z))	2.3*	Glycerophospholipids

Significance (Students *t*-test): *P < 0.05, **P < 0.01, ***P < 0.001.

RRI = Resistance related induced metabolites [(RP/RM)/(SP/SM)], R & S are resistant and susceptible genotypes, P & M are pathogen or mock inoculated; PA = Phosphatidic acid; FC = Fold-change of RRI metabolites; HCAA = Hydroxycinnamic acid amide.

**Table 2:** Promoter sequence analysis of resistance related metabolite biosynthetic genes (*R*_*RRM*_) regulated by the transcription factor *TaWRKY70*.

Genes	GenBank accession no.	W-BOX sequence	Position (bp)
*TaACT*	KT962210	TCGCTGGTGATGACTAGAGGCGGCC	−464
*TaDGK*	KU562862	ATTATACTTATTGACTTTGCATCAAG	−281
*TaGLI1*	KC244204	GTGATAGTCATTGACTTCCACGCCCA	−250

*TaACT* = *T. aestivum* agamatinecoumaroyl transferase gene; *TaDGK* = *T. aestivum* diacylgycerol kinase gene; *TaGLI1* = *T. aestivum* glycerol kinase gene. Position = is the localization of W-box region upstream to the ATG start site.
