# Supplementary material for: TaWRKY70 transcription factor in wheat QTL-2DL regulates downstream metabolite biosynthetic genes to resist Fusarium graminearum infection spread within spike
Source: Sci Rep. 2017 Feb 15;7:42596. doi: 10.1038/srep42596 (PMC5309853; doi:10.1038/srep42596)
Supplement: Supplementary Dataset [file srep42596-s1.doc]

**TaWRKY70 transcription factor in wheat *QTL-2DL* regulates downstream metabolite biosynthetic genes to resist *Fusarium graminearum* infection spread within spikelets**

Udaykumar Kage, Kalenahalli N. Yogendra and Ajjamada C. Kushalappa*

**Figure S1:** Fragment design for VIGS. A) Primers designed for knocking down *TaGLI1* gene. Virus-induced gene silencing (VIGS) fragments were designed to specifically knock-down the *TaGLI1* gene. The knock-down fragment is boxed; b) Representation of typical BSMV based VIGS vectors and c) The *TaWRKY70*cDNA fragment was cloned to pSL038-1 vector (Test, in the top) downstream of the γb gene. pSL038-1 vector carrying either *phytoene desaturase* (*PDS*) gene (BSMV:*TaPDS,* in the middle) or without any plant gene (BSMV:00, in the down) served as positive control and negative controls, respectively.


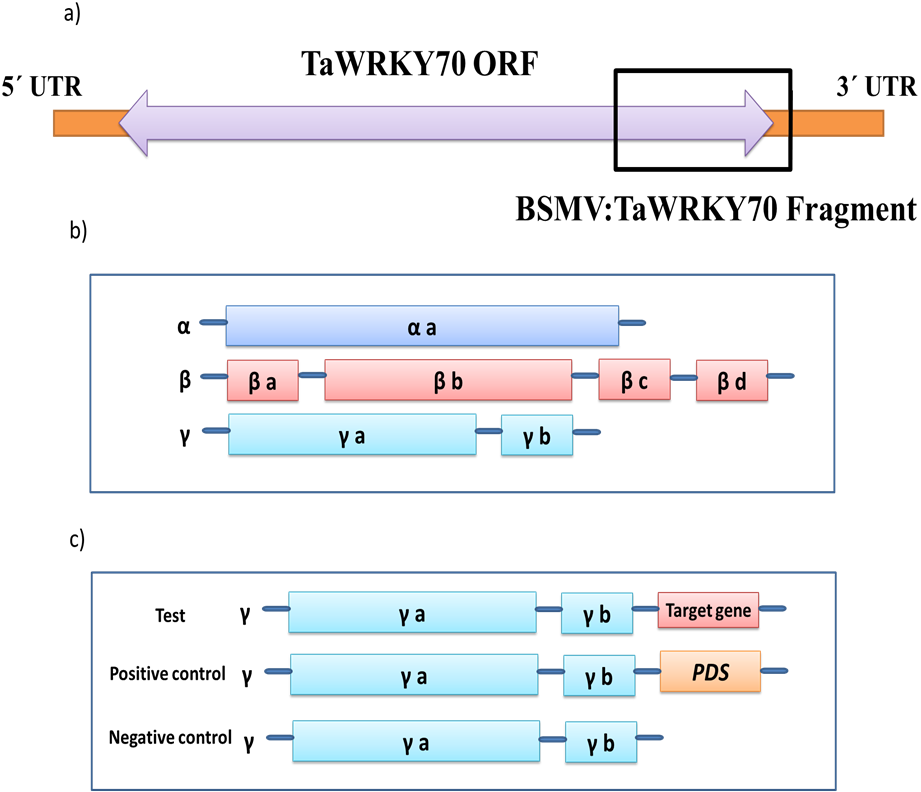


**Figure S2:** In-silico DNA-protein interaction using GeneMANIA server. Here dark colored rounds indicate target genes, DGK8 (TaDGK), HCT (TaACT), WRKY70 (TaWRKY70) and NHO1 (TaGLI1).

**Figure S3:** Photobleaching symptoms on wheat spikes observed at 12 days inoculated with BSMV:*TaPDS* and BSMV:00 vectors.


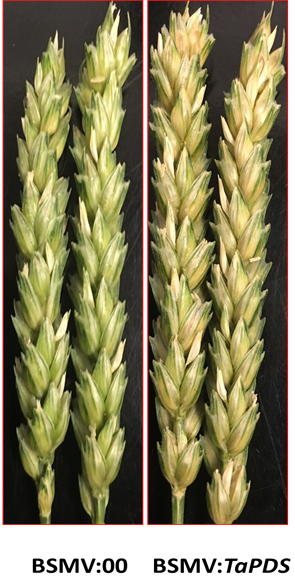


**Figure S4:** A proposed model showing *TaWRKY70* regulating downstream genes involved in the biosynthesis of hydroxycinnamic acid amides (HCAAs) and phosphotidic acid and derivatives (PAs) to resist the pathogen through cell wall fortification, intensified signaling and reduced cell death. After pathogen perception, *TaWRKY70* gets activated by unknown pathways (ex: MAP kinase) and this intern regulates the transcript expression of downstream genes involved in biosynthesis of resistance related induced (RRI) metabolites.

**
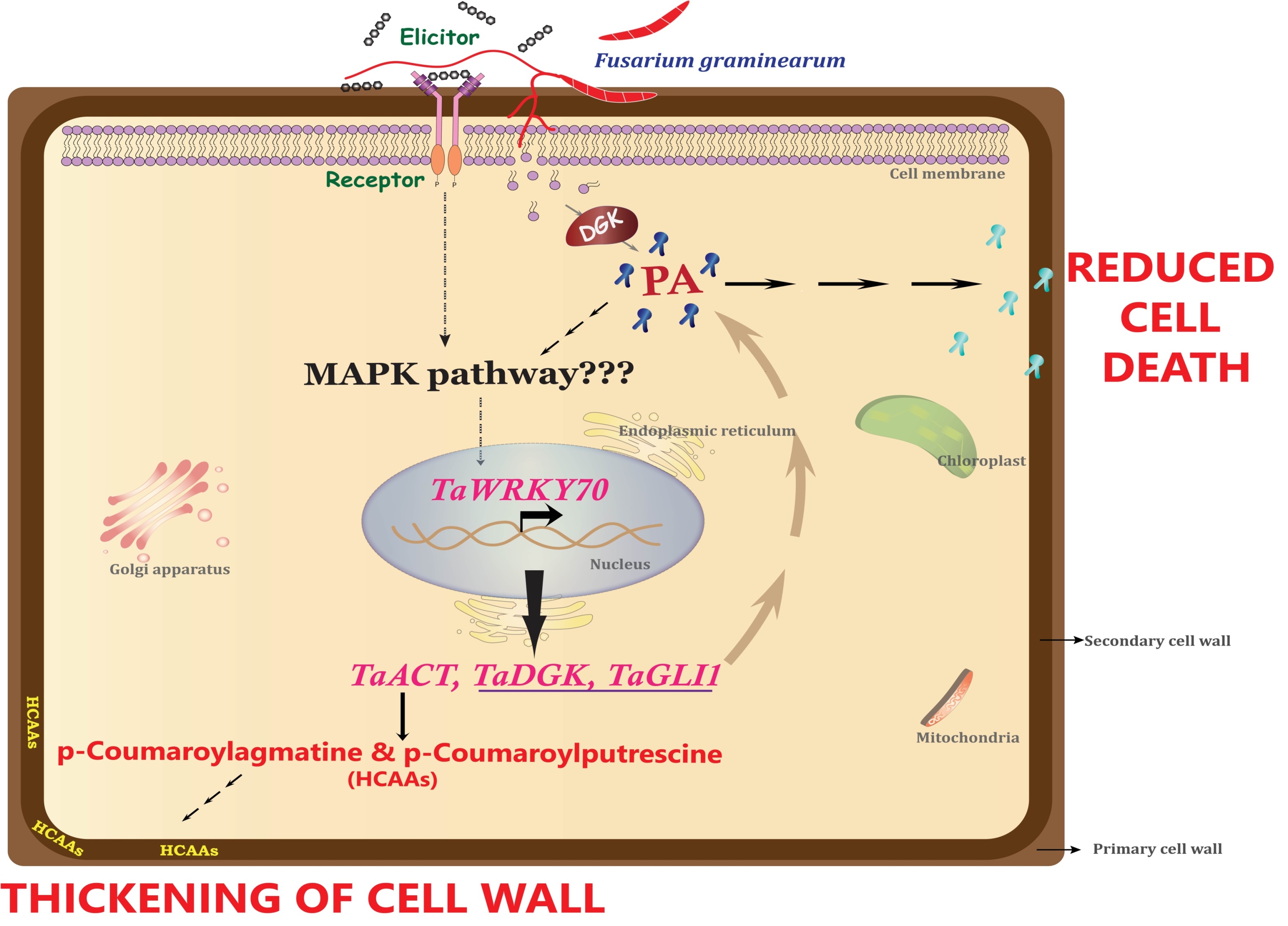
**

**Table S1: List of primers used in this study.**

| **Table S1: List of primers used in the experiments** | | | |  |
| --- | --- | --- | --- | --- |
|  |  | **Primers** | |  |
|  | **Name** | **Forward** | **Reverse** |  |
| **Gene sequencing** | *TaWRKY* | GGAGCAGGAGAGTGTTCGAG | CAACCGGGAAGATCGAAGAT |  |
| **VIGS Fragment amplification** | *TaWRKY_VIGS* | CGCATGCTCCTCACCTCACCGG | CCGTCGCAGCAGCGTCGTCGA |  |
| **VIGS gene expression** | *TaWRKY-VIGSqPCR* | TCGACGACGCTGCTGCGACG | GCAGAGCTCCCTGGCGTGC |  |
| **Gene sequencing** | *TaDGKq* | CGGCCCGCATGACTTGCT | TGGAATGCGGTAGTCCGACA |  |
|  | *TaGLI1q* | TCAAGCAGCACTACCCG | CAAACCAGCATCCACATTA |  |
|  | *TaWRKYqPCR* | TCGACGACGCTGCTGCGACG | GCAGAGCTCCCTGGCGTGC |  |
|  | *TaACTqPCR* | ACCACGCGATCCGCCGCGAG | CGGCGTGGCGTTCGTCGTCGTT |  |
|  | *TaACTIN* | ACCTTCAGTTGCCCAGCAAT | CAGAGTCGAGCACAATACCAGTTG |  |
|  | *Tri6* | TCTTTGTGAGCGGACGGGACTTTA | TGTTGGTTTGTGCTTGGACTCAT |  |
| **Promoter sequencing** | *TaACTp* | GCCGCCACCCAGATCCAATC | GGCGACAAGTGCAAGGTTA |  |
|  | *TaDGKp* | GTTGCCGTGTTGCCGAGGGT | GCCAGAACCGTTAGAACCAATTGC |  |
|  | *TaGLI1p* | TCAGGTCAATTGGACTCCGTTTG | CTGGGAAGCAGTTGGGAGCGG |  |
| **NLS Assay** | *TaWRKYNLS* | ATCCCCAATACTATGTCCATGGCG | GATCCCCAATACTCAATGGTCGAG |  |
| **LUC Assay** | *WRKY_LUC* | GAATCCATGTCCATGGCGCCGTACGAG | GAGCTCCTGCTCAGCACCTCCTCCT |  |
|  | *ACT_LUC* | CTCGAGGCCGCCACCCAGATCCAATC | GCGGATCCGGCGACAAGTGCAAGGTTA |  |
|  | *DGK_LUC* | CTCGAGGTTGCCGTGTTGCCGAGGGT | GGATCCGCCAGAACCGTTAGAACCAATTGC |  |
|  | *GLI1_LUC* | CTCGAGTCAGGTCAATTGGACTCCGTTTG | GGATCC CTGGGAAGCAGTTGGGAGCGG |  |

**Table S2: Predicted genes present in the QTL-2DL based on synteny with Brachypodium and Rice.**

|  |  |  |  |
| --- | --- | --- | --- |
| **S.N** | **Gene on chromosome 4 of Rice** | **Gene ID** | **Wheat scaffold** |
| 1 | Cysteine-rich receptor-like protein kinase 7 precursor, putative | LOC_Os04g01860 | IWGSC_chr2DL_ab_k71_contigs_longerthan_200_9734312 |
| 2 | Dihydrodipicolinate synthase, chloroplast precursor, putative, expressed | LOC_Os04g18200.1 | IWGSC_chr2DL_ab_k71_contigs_longerthan_200_9763750 |
| 3 | ABC transporter - CER5 | EU127477.2 | IWGSC_chr2DL_ab_k71_contigs_longerthan_200_9821167 |
| 4 | Phenylalanine ammonium transferase - PAL | LOC_Os04g43800.1 | IWGSC_chr2DL_ab_k71_contigs_longerthan_200_9823366 |
| 5 | Chloroplast 30S ribosomal protein S7, putative | LOC_Os04g16712.1 | IWGSC_chr2DL_ab_k71_contigs_longerthan_200_9827360 |
| 6 | Photosynthetic reaction center protein, putative, expressed | LOC_Os12g19580.1 | IWGSC_chr2DL_ab_k71_contigs_longerthan_200_9831608 |
| 7 | Glycerol-3-phosphate acyltransferase - GPAT3 | EMT04881.1 | IWGSC_chr2DL_ab_k71_contigs_longerthan_200_9834552 |
| 8 | Translation initiation factor IF-1, chloroplast, putative | LOC_Os04g16834.1 | IWGSC_chr2DL_ab_k71_contigs_longerthan_200_9837111 |
| 9 | NADPH-dependent oxidoreductase, putative, expressed | LOC_Os12g12470.1 | IWGSC_chr2DL_ab_k71_contigs_longerthan_200_9853575 |
| 10 | Photosystem II reaction center protein H, putative | LOC_Os04g16848.1 | IWGSC_chr2DL_ab_k71_contigs_longerthan_200_9843343 |
| 11 | Serine/threonine-protein kinase receptor precursor, putative, expressed | LOC_Os04g01310.1 | IWGSC_chr2DL_ab_k71_contigs_longerthan_200_9860267 |
| 12 | Photosystem II reaction center protein K precursor, putative, expressed | LOC_Os10g21198.1 | IWGSC_chr2DL_ab_k71_contigs_longerthan_200_9864032 |
| 13 | Protein kinase, putative, expressed | LOC_Os04g01874.1 | IWGSC_chr2DL_ab_k71_contigs_longerthan_200_9865552 |
| 14 | Pectin lyase-like superfamily protein | LOC_Os04g52320.1 | IWGSC_chr2DL_ab_k71_contigs_longerthan_200_9714859 |
| 15 | Cell wall invertase 2 | LOC_Os04g33740.1 | IWGSC_chr2DL_ab_k71_contigs_longerthan_200_9733491 |
| 16 | Succinate dehydrogenase 5 | Bradi5g09750.1  LOC_Os04g34100.1 | [IWGSC_chr2DL_ab_k71_contigs_longerthan_200_9821901](http://plants.ensembl.org/Triticum_aestivum/Location/View?g=Traes_2DL_1751C5EF1;r=IWGSC_CSS_2DL_scaff_9821901:2483-3918:-1) |
| 17 | Auxin signaling F-box 2 | Bradi5g08680.1 | [IWGSC_chr2DL_ab_k71_contigs_longerthan_200_9848890](http://plants.ensembl.org/Triticum_aestivum/Location/View?g=Traes_2DL_F0FEB5383;r=IWGSC_CSS_2DL_scaff_9848890:1-4145:1) |
| 18 | NB-ARC domain-containing disease resistance protein | Bradi5g15560.1  LOC_Os04g43440.1 | [IWGSC_chr2DL_ab_k71_contigs_longerthan_200_9861014](http://plants.ensembl.org/Triticum_aestivum/Location/View?g=Traes_2DL_D4AAE1E4C;r=IWGSC_CSS_2DL_scaff_9861014:965-4907:-1) |

***Gene ID containing:** LOC – Rice, Bradi – Brachypodium, others - wheat
